# Bilateral cystoid macular edema following docetaxel chemotherapy in a patient with retinitis pigmentosa: a case report

**DOI:** 10.1186/s12886-015-0020-4

**Published:** 2015-03-29

**Authors:** Anna Enzsoly, Kinga Kammerer, Janos Nemeth, Miklos Schneider

**Affiliations:** Department of Ophthalmology, Semmelweis University, Faculty of Medicine, H-1085 Maria u. 39, Budapest, Hungary; Department of Oncology, Flor Ferenc Hospital of Pest County, Kistarcsa, Hungary

**Keywords:** Docetaxel, Cystoid macular edema, Optical coherence tomography, Retinitis pigmentosa

## Abstract

**Background:**

Docetaxel is a chemotherapeutic agent of the taxane class of drugs for the treatment of breast cancer. We present a female patient who noted decreased vision after docetaxel treatment.

**Case presentation:**

A 45-year-old female patient received docetaxel treatment after resection of a breast carcinoma. Funduscopy and optical coherence tomography (OCT) showed cystoid macular edema on both eyes. Dilated funduscopy also showed bone spicule-like pigmented deposits, typical for retinitis pigmentosa. Besides the fundus appearance restricted peripheral vision and scotopic electroretinogram confirmed the diagnosis of retinitis pigmentosa. Chemotherapy was discontinued following a consulation with the oncologist of the patient. After five weeks, visual acuity improved significantly along with decrease of retinal thickness measured by OCT.

**Conclusion:**

Docetaxel may cause ocular adverse effects such as cystoid macular edema. Ophthalmological examination is warranted for patients with visual complaints during docetaxel chemotherapy.

## Background

Docetaxel belongs to taxane class of drugs, which are anti-mitotic chemotherapeutical agents registered for the treatment of various types of solid tumors including breast cancer [1].

Retinitis pigmentosa (RP) is an inherited retinal dystrophy causing progressive visual field constriction. RP initially affects midperipheral photoreceptors, then as the disease progresses more central retinal regions become affected. Typically, structural changes such as decrease of the foveal thickness and retinal pigment epithelium atrophy and functional changes on full-field and multi-focal electroretinogram (ERG) are associated with RP [2,3]. Cystoid macular edema (CME) can be detected in 10–20% of RP patients [4].

We present a female patient who noted decreased vision after docetaxel treatment.

## Case presentation

A 45-year-old Caucasian woman received docetaxel-doxorubicin-cyclophosphamide chemotherapy after surgical resection of an invasive ductal breast carcinoma in clinical stage IIb.

According to the medical history of the patient, she had no pre-existing ocular diseases, and based on her statement her ocular symptoms started following the beginning of the treatment. She presented at our department 12 weeks after initiation of chemotherapy. By that time she already received 4 series of TAC treatment (docetaxel 75 mg/m^2^, doxorubicin 50 mg/m^2^ and cyclophosphamide 500 mg/m^2^ q3w).

Her best corrected visual acuity (BCVA) was 0.3 and 0.2. Anterior segments showed no pathologies, intraocular pressure was normal.

**Figure 1:**
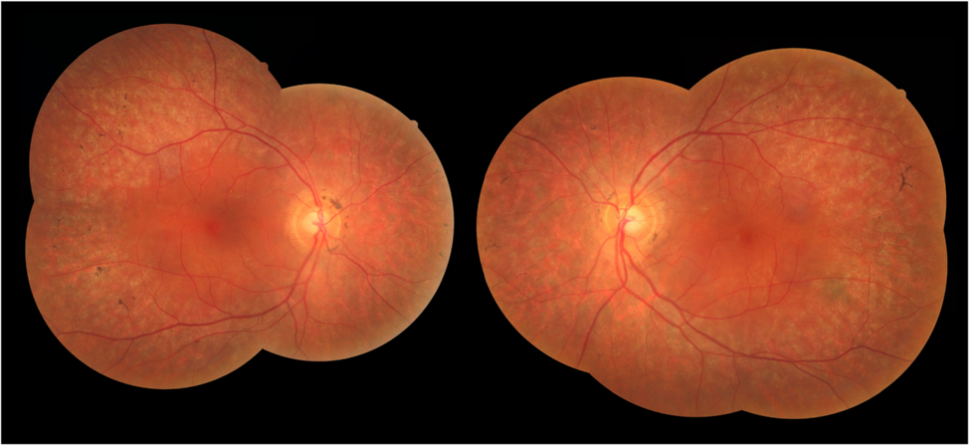
**Composit fundus photography.** Bone spicule-like pigmented deposits at the mid-periphery and cystoid macular edema at the fovea symmetrically on both sides.

**Figure 2:**
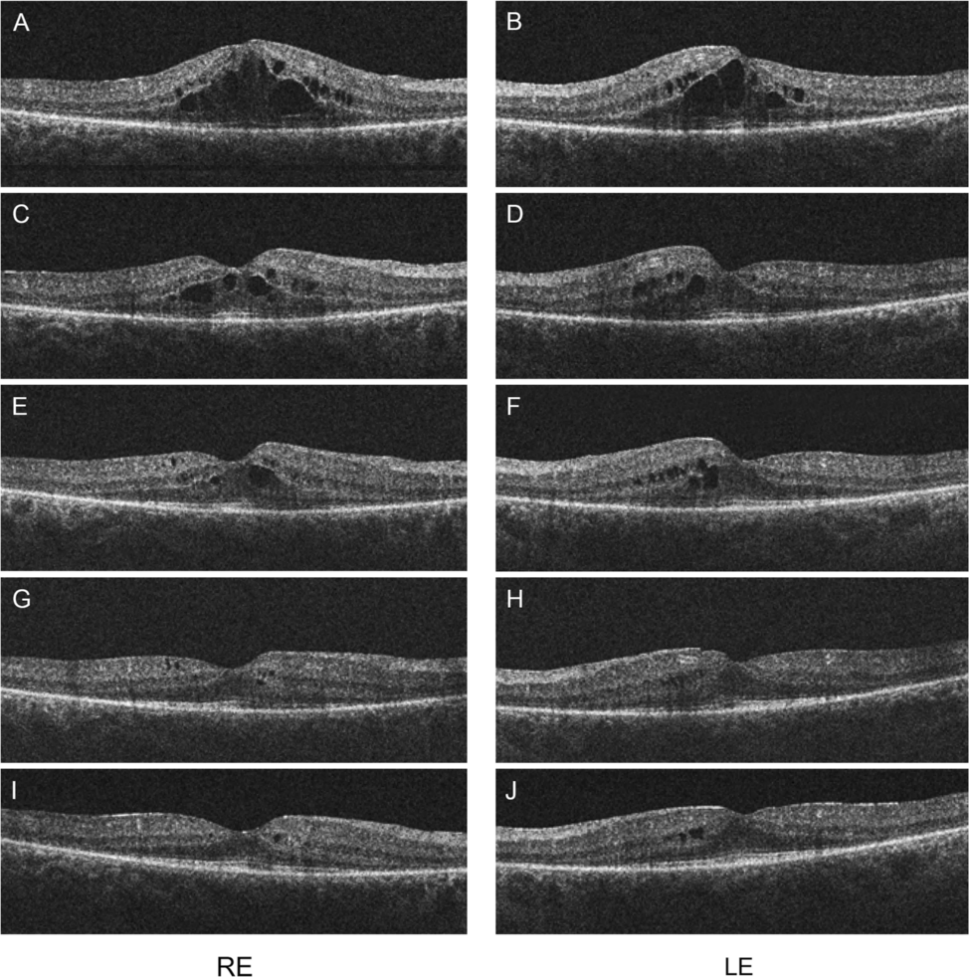
**OCT scans of the macula of both eyes after 4 series of TAC treatment (A, B).** Large cystic spaces are present in the outer nuclear layer and small cystic spaces in the inner nuclear layer. Photoreceptor inner/outer segment (IS/OS) junction is discontinuous but relatively intact underneath the fovea and absent outside this area. Retinal thickness is lower outside the perifoveal area. Scans 5 weeks **(C, D)**, 8 weeks **(E, F)**, 12 weeks **(G, H)** and 17 weeks **(I, J)** after discontinuation of the chemotherapy. Retinal thickness in the perifoveal area gradually decreased along with the resolution of the intraretinal cystoid spaces.

Dilated fundus examination revealed cystoid macular edema (CME) around the fovea on both eyes (Figure 1), which was also confirmed by optical coherence tomography (OCT) (Figure 2A and B). Large cystic spaces were observed in the outer nuclear layer and small cystic spaces in the inner nuclear layer on both eyes. Photoreceptor inner/outer segment (IS/OS) junction was discontinuous but relatively intact underneath the fovea and absent outside this area. Retinal thickness was increased in the center of the macula according to the edema and subnormal outside the perifoveal area. There was no evidence of vitreo-retinal interface, retinal pigment epithelium (RPE) or sub-RPE abnormalities on the images.

Additionally, as an unexpected finding bone spicule-like pigmented deposits could be seen at the periphery, characteristic for retinitis pigmentosa (RP) (Figure 1). Scotopic electroretinogram was almost completely extinguished and visual field showed concentric constriction to 5° on both eyes, confirming the presumed diagnosis.

Fluorescein angiography probably would not have had an effect on differential diagnosis as no leakage was expected based on the literature of CME induced by either RP or taxane-based chemotherapy [5-7]. Kuznetcova et al. [7] suggest that the mechanisms of developing CME by certain etiologies, e.g. inflammatory diseases and taxane treatment may be different. In CME cases induced by inflammation the tight junctions are affected and they become potential leaking points. Taxane treatment causes dysfunction in the cytoskeleton of the retinal pigment epithelium, the choroid - pigment epithelium border is unaffected and therefore leakage is not to be expected. Besides lacking differential diagnostic value, results of the angiography would not have had any therapeutic consequences either. Taking these facts to consideration and judging the general clinical state of the patient fluorescein angiography was not performed.

At this point, following a consultation with the patient’s oncologist and detailed discussion with the patient, chemotherapy was stopped. Five weeks after the discontinuation of chemotherapy and additional topical nepafenac treatment the visual acuity of the patient improved significantly. After 4 months of follow-up BCVA was 1.0 on both eyes. Retinal thickness in the perifoveal area also decreased gradually with the resolution of intraretinal cysts on OCT scans (Figure 2G and H). Visual acuity and retinal thickness remained stable after discontinuation of the topical therapy.

## Discussion

Our patient presented with bilateral CME and RP after receiving docetaxel-doxorubicin-cyclophosphamide treatment. The most likely explanation for these conditions is that CME was a chemotherapy side-effect while RP was an undetected pre-existing disease. This association is also supported by the reversibility of CME after discontinuation of chemotherapy. Taxane-based chemotherapy may induce CME as reported by more than a dozen case studies in the past decade [5,6,8-11]. The majority of these studies concern paclitaxel treatment. There are only two case studies that report findings on a CME after docetaxel treatment. In the first Teitelbaum and coworkers [6] describe a CME with normal fluorescein angiography diagnosed by fundoscopy, in the second by Telander et al. [11] diagnosis is backed by OCT evidence. There are no reports so far in the literature of a co-occurrence of RP and docetaxel induced CME. According to papers dealing with CME induced by paclitaxel, outer retinal layers seem to be more affected [12]. However, both docetaxel treatment and RP associated CME are reported to involve inner and outer layers [7,13], this is also the case with our patient.

To our knowledge, no cases are reported that CME may be caused by cyclophosphamide or doxorubicin treatment, however the combination of cyclophosphamide and paclitaxel was reported in one CME case [5].

There are three alternative hypotheses: First, based on our observations, it is possible that docetaxel treatment may trigger the progression of a pre-existing RP and also increase the likelihood for CME to develop. Chaudhry et al. reported that in patients treated with chemotherapy, neuropathy may develop early or existing neuropathy may get worse in cases of pre-existing neuropathology, e.g. diabetes mellitus [14]. According to this study, in our case RP could have acted as a pre-existing neuropathology and chemotherapy as a trigger that caused the disease to progress to be symptomatic.

Second is the possibility that RP may also have been a consequence of chemotherapy, even though we consider this explanation highly unlikely. We are not aware of any chemotherapeutical agents proven to be associated with the development of RP, there is only one case study reporting acceleration of RP after chemotherapy in a patient with non-Hodgkin’s lymphoma [15]. As we have no previous ophthalmological documentation of the patient, it is not known whether RP existed prior to the chemotherapy. It is possible that in this case RP has developed due to a neurotoxic effect of any component of the patient’s chemotherapeutical regimen. Neurotoxic side effects of taxanes are frequently recognized, ocular lesions like alterations on the electroretinogram can also occur [16]. The mechanism of taxanes is to stabilize microtubules to stop their mitotic activity. This mechanism of action is suggested to be associated with their neurotoxic side effects as microtubules are responsible for intracellular transport and other critical cell functions of neurons [17] including photoreceptors [18]. In a recent study, it was reported that in RP the extent of hypoautofluorescent parafoveal arc may correspond with the duration of the disease [19], although prospective longitudinal studies have not confirmed this observation yet. Unfortunately, we do not have access to wide-field fundus autofluorescence, thus this second explanation of short-term RP cannot be ruled out this way.

Finally, we cannot exclude another unlikely possibility that the patient developed CME as a complication of her RP exclusively. Since CME can occur at any stage of the disease [4] the association with chemotherapy may be coincidental, but the reversibility of the edema after chemotherapy was stopped suggests otherwise.

Another important issue to discuss is the termination of the anti-cancer treatment once an ophthalmic side-effect occurs. In our case the decision was simple as the patient underwent complete surgical resection and only had one cycle of TAC left of her final chemotherapy treatment. In other cases the ophthalmologist and the oncologist should weigh the risks and benefits of chemotherapy cessation, but the decision should ultimately be left at the discretion of the oncologist.

## Conclusions

In conclusion chemotherapy treatment including docetaxel may cause ocular adverse effects such as cystoid macular edema. Further studies are required to reveal the possible retinal neurotoxicity of taxanes. It might also be worthwhile to investigate neuroprotective agents in combination with chemotherapy.

Our observations draw the attention towards possible ophthalmological side-effects of docetaxel chemotherapy. It is important for oncologists to advise patients of the possibility of treatment-related visual complaints and to report those. Ophthalmic evaluation is warranted to patients with such complaints.

## Consent

This study was conducted in accordance with the ethical standards stated in the Declaration of Helsinki. The patient was fully informed about the examinations, and provided written consent. Written consent was obtained from the patient for publication of this material. A copy of the consent is available for review.
